# Somato-Psychic Pathway: a universal developmental trajectory linking somatic structural–functional integrity, autonomic regulation, and the emergence of mind

**DOI:** 10.3389/fnint.2026.1771123

**Published:** 2026-03-02

**Authors:** Boris Živný

**Affiliations:** NeuroCentrum Clinic, SPAD Institute, Jesenice, Czechia

**Keywords:** autonomic dysregulation, autonomic regulation, developmental psychopathology, embodied cognition, interoception, neurodevelopment, Somato-Psychic Pathway

## Abstract

The Somato-Psychic Pathway (SPP) is proposed as a universal ontogenetic developmental trajectory through which somatic structural–functional integrity and autonomic regulation shape the emergence and stability of the mind under both physiological and pathological conditions. Integrating insights from developmental neuroscience, evolutionary biology, and clinical neurodevelopment, SPP conceptualizes mental functions as interpretive extensions of bodily and autonomic states rather than as their primary generators. The framework delineates a developmentally constrained directional sequence beginning with somatic organization, proceeding through proprioceptive and interoceptive accuracy, and culminating in autonomic regulation, emotional stability, and cognitive–social maturation. Disruption of this trajectory—most prominently through axial dysfunction, distorted joint–muscle–fascial proprioception, persistent low-grade nociceptive drive, or direct mechanical influences on peripheral autonomic structures—is proposed to lead to Somato-Psychic Autonomic Dysregulation (SPAD), a state characterized by chronically reduced autonomic flexibility and heightened threat responsivity. Prolonged operation of the pathway in this pathological mode gives rise to the clinical phenotype termed Somato-Psychic Syndrome (SPS). The SPP framework emerged from longitudinal clinical observation of disrupted and restituted developmental trajectories, providing a unique ontogenetic perspective on the directionality of neurodevelopmental regulation. By integrating the somatic, autonomic, emotional, and cognitive domains into a single regulatory continuum, SPP offers a biologically grounded model with implications for understanding childhood neurodevelopmental disorders and guiding future therapeutic strategies.

## 1 Introduction

The development of the human mind does not begin with cognition. It begins with the body. Long before cortical networks mature, the infant engages with the world through developmentally evolving fluctuations of muscle tone, gravitational orientation, visceral rhythms, autonomic states, and a proprioceptive field that anchors every movement and early interaction (Thelen, 2004; Smith and Gasser, 2005). What later comes to be thought initially exists as patterning within physiological experience: changes in breathing, shifts in arousal, transitions between comfort and distress, and variations in postural readiness that are continuously shaped by bodily and autonomic signals (Craig, 2002; Critchley and Harrison, 2013).

Despite this embodied foundation, clinical neurodevelopment has traditionally approached childhood difficulties from psychological, behavioral, or cortex-centric perspectives. Influential models address perception, affect, or cognition, yet few frameworks integrate somatic structural–functional organization, proprioceptive and nociceptive signalling, autonomic regulation, and higher psychological functions into a single ontogenetic developmental sequence. The Somato-Psychic Pathway (SPP) is proposed as such a sequence. It conceptualizes psychological development as scaffolded by bodily organization and autonomic stability and argues that deviations from these foundations produce predictable patterns of emotional, sensory, behavioral, and cognitive disturbance.

SPP is not presented metaphorically but as a developmentally structured framework describing a hierarchical ontogenetic trajectory that operates under physiological conditions in typical development and under pathophysiological conditions when disrupted. The framework originated in longitudinal clinical observation. Across children with complex neurodevelopmental profiles, a recurring directional pattern was observed: somatic disruption preceded autonomic dysregulation, which in turn preceded emotional instability, sensory hypersensitivity, attentional fluctuation, and communicative or cognitive delay. When somatic integrity was restored, improvement unfolded in the reverse sequence. This reversibility was interpreted as consistent with an inherent developmental directionality of the underlying mechanism and supported the crystallization of the general principles of SPP.

### 1.1 Epistemological status and scope of the Somato-Psychic Pathway

The Somato-Psychic Pathway (SPP) is proposed as a developmentally oriented heuristic framework derived from longitudinal clinical observation and theoretical synthesis across developmental neuroscience, autonomic physiology, embodied cognition, and systems-based models of neurodevelopment. It is not presented as a finalized mechanistic theory nor as an empirically confirmed causal account. Rather, SPP is articulated as a structured and testable developmental hypothesis concerning directional organization across somatic, autonomic, affective, and cognitive domains.

The framework advances the concept of developmental priority rather than deterministic causation. Specifically, SPP proposes that somatic structural–functional organization and autonomic regulation constitute developmentally upstream regulatory layers that constrain and shape the emergence, stability, and flexibility of emotional and cognitive functions. This directional formulation does not deny reciprocal or bidirectional influences between levels of organization. Contemporary neuroscience clearly demonstrates circular interactions between bodily states, autonomic regulation, emotional experience, and cognitive appraisal. However, SPP posits that, within ontogenetic development, somatic and autonomic processes typically establish the regulatory conditions within which higher psychological functions stabilize and mature. Developmental priority, in this sense, refers to scaffolding rather than to linear one-way determination.

Within the SPP framework, the term “universal” denotes a developmentally constrained organizing principle rather than an invariant developmental pathway. The concept does not imply that all forms of psychopathology originate in somatic disturbance, nor that all developmental trajectories strictly conform to a single rigid sequence. Instead, “universal” refers to the proposition that embodied and autonomic regulation constitute necessary developmental substrates for stable psychological organization across both typical and atypical development. Individual variability, cultural influences, neurodiversity, environmental conditions, and primary genetic or central nervous system pathologies may interact with, modify, or override the pathway in complex ways. These factors do not invalidate the framework but delineate its boundary conditions.

SPP emerged from recurrent longitudinal clinical observations in children presenting with heterogeneous neurodevelopmental profiles. Across these observations, a consistent directional pattern was noted: somatic disruption frequently preceded autonomic dysregulation, which in turn preceded emotional instability and cognitive or communicative delay. Conversely, clinical improvement often unfolded in a reverse sequence, with autonomic stabilization preceding affective and cognitive gains. These patterns are interpreted as inferentially consistent with the proposed developmental ordering. Nevertheless, such observations remain hypothesis-generating rather than confirmatory and require systematic empirical investigation under controlled conditions.

Accordingly, SPP is best understood as a biologically grounded developmental scaffold intended to organize clinical phenomena and generate empirically tractable predictions. It does not replace existing diagnostic frameworks, nor does it claim exclusive explanatory status. Instead, it provides an integrative developmental lens linking somatic organization, autonomic regulation, emotional stability, and cognitive maturation within a single regulatory continuum. Its scientific value lies in its capacity to articulate explicit developmental hypotheses, specify mediating mechanisms, define boundary conditions, and stimulate longitudinal and interventional research designed to examine the proposed ordering and its clinical implications.

## 2 The primacy of the body in development

Across evolution and ontogenetic development, the body and autonomic systems form the foundation on which complex neural functions are built. Brainstem circuits, vestibular systems, primitive sensorimotor loops, and the autonomic nervous system establish the foundational regulatory conditions necessary for cortical development and continue to modulate cortical function across late development and throughout the lifespan (Critchley and Harrison, 2013; Porges, 2007). Emotional life begins as differentiation in physiological state—alterations of vagal tone, sympathetic activation, muscular bracing, and respiratory synchrony—long before conceptualized emotions or symbolic cognition emerge (Craig, 2002; Seth, 2013).

These physiological patterns constitute the earliest units of affective organization. Through repeated interaction with the environment, they consolidate into the foundations of attachment, exploration, and self-regulation (Smith and Gasser, 2005; Thelen, 2004). Cognitive and linguistic capacities subsequently develop from the stability provided by these lower-level systems. A stable autonomic regulatory baseline supports curiosity, learning, and social engagement, whereas autonomic instability constrains exploratory behavior and narrows developmental possibilities (Beauchaine, 2015; Thayer et al., 2012).

Within this perspective, the body does not merely respond to the mind. Bodily and autonomic dynamics create the developmental landscape in which the mind can emerge. Psychological maturation is therefore inseparable from the bodily and autonomic conditions that support it.

## 3 Somatic structural–functional integrity: peripheral autonomic mechanisms and proprioceptive, interoceptive, and nociceptive inputs

Somatic structural–functional integrity encompasses the dynamic organization of axial and appendicular musculoskeletal structures, their mobility, alignment, muscular coordination, fascial tensioning and strain patterns, and the mechanical, metabolic, and humoral environment surrounding peripheral autonomic pathways. This integrity is communicated to the nervous system through multiple physiological channels, of which three afferent modalities are central to SPP: proprioceptive, interoceptive, and nociceptive signalling. In parallel, somatic structural–functional integrity may directly modulate autonomic regulation through local mechanical influences on its peripheral structures.

Proprioception provides a continuous multisensory mapping of the body in space. Muscle spindles, Golgi tendon organs, joint receptors, ligamentous and fascial mechanoreceptors, and micro-movements across intervertebral segments generate a layered, moment-to-moment representation of posture and movement (Proske and Gandevia, 2012). Proprioception is fundamental not only for motor accuracy but also for postural control, body schema formation, and adaptive interaction with the environment throughout ontogenetic development. When axial structures lose physiological mobility or symmetry, this mapping becomes distorted. This state is referred to as Distorted Proprioceptive Information (DPI), reflecting a mismatch between peripheral somatic input and the central nervous system’s predictive models of posture and movement. Inconsistent proprioceptive input, as captured by DPI, disrupts vestibular calibration, ocular-motor control, anticipatory postural strategies, and the stability of sensorimotor loops, thereby tending to bias autonomic regulation toward rigidity and threat monitoring.

In parallel with proprioceptive signalling, interoceptive afference conveys information about the internal physiological state of the body, including visceral tension, respiratory and cardiovascular rhythms, and metabolic load, thereby providing a continuous internal context that interacts with both proprioceptive and nociceptive inputs in shaping autonomic regulation. Beyond skeletal muscles, ubiquitous fascial tissues form a continuous, body-wide sensory network that predominantly provides modulatory somatosensory afferent input, encompassing nociceptive, interoceptive, and—more modestly—proprioceptive components. While this system exhibits a degree of regional topographic organization, its afferent signalling is largely diffuse and low-resolution, serving primarily to modulate central autonomic nervous system state rather than to convey discrete sensory content. Consequently, fascial afference influences psychological responses only indirectly, via its impact on central autonomic regulation (Benjamin, 2009; Langevin, 2021; Porges, 2023).

A third major mechanism involves persistent nociceptive input, often low-grade and easily overlooked. Many axial disturbances generate nociceptive signalling even in the absence of consciously perceived pain. While high-intensity nociceptive input can acutely disrupt autonomic regulation, persistent low-grade nociception is often sufficient to induce comparable autonomic dysregulation through cumulative and largely unnoticed effects. Irritated zygapophyseal joints, strained paraspinal musculature, ligamentous micro-irritation, dural tension, and altered load distribution can all contribute to a persistent nociceptive drive. Neuroimaging and systems-level studies demonstrate that nociceptive input engages distributed networks implicated in autonomic output, affect regulation, and salience detection (Tracey and Mantyh, 2007; Baliki and Apkarian, 2015). Within spinal and brainstem circuits, nociceptive and proprioceptive channels converge. When nociceptive input becomes persistent, these circuits are sensitized, and autonomic regulation is biased toward defensive readiness, even in the absence of overt injury.

An additional, often overlooked component involves direct mechanical influences on peripheral autonomic structures, conveyed through autonomic afferent fibres and ganglia rather than through somatosensory pathways, and further shaped by local metabolic and humoral conditions within surrounding tissues. The sympathetic chain, vagal branches, and autonomic plexuses course through regions that are mechanically coupled to the axial skeleton, rib cage, diaphragm, and cervical connective tissues. Altered mobility, sustained muscular tension, or fascial densification can therefore directly modulate autonomic signalling. Experimental and clinical evidence indicate that mechanical stimulation or restriction of spinal and paraspinal tissues alters sympathetic outflow, heart rate variability, and visceral function (Pickar, 2002; Budgell and Polus, 2006). These proposed peripheral mechanical influences should be understood as probabilistic modulatory factors rather than deterministic causal mechanisms and require targeted empirical verification.

Together, distorted proprioception, altered interoceptive signalling, persistent nociceptive input—whether suprathreshold or subthreshold—and direct modulation of peripheral autonomic structures constitute a continuous somatic drive. When this drive exceeds adaptive capacity, the Somato-Psychic Pathway shifts from physiological integration into pathological operation.

Mechanical influences arising from the periphery may affect autonomic regulation through two distinct yet complementary, directionally convergent pathways activated by the same peripheral mechanical inputs. One pathway involves direct mechanical modulation of peripheral autonomic structures, whereby altered tissue tension, mobility, or spatial relationships locally influence autonomic ganglia, fibres, and plexuses. This mechanism operates at the peripheral level and may contribute both to pathological autonomic dysregulation and, when applied deliberately, to therapeutic modulation, typically with relatively predictable effects on autonomic regulation and its mental consequences. The second pathway operates indirectly: the same mechanical influences are transduced into proprioceptive, interoceptive, and nociceptive afferent signals, which are centrally integrated and subsequently modulate autonomic centres in the brain. Therapeutic engagement of this indirect, afferent-mediated pathway is inherently less specific and more challenging to control. Nevertheless, it is inevitably co-activated during mechanical interventions applied outside an explicit autonomic target. Its contribution, therefore, represents a clinically relevant side effect that must be considered in diagnostic reasoning and in the conceptual planning of therapeutic strategies, even when autonomic modulation is not the primary therapeutic intent. In physiological and clinical contexts, these pathways operate in parallel and in the same causal direction—from peripheral bodily influences to central autonomic regulation and downstream psychological responses. The relative contribution of each pathway depends primarily on the anatomical location of the mechanical influence and on the specific neural structures engaged at that site.

## 4 Autonomic regulation as the central integrative layer

The autonomic nervous system constitutes the functional nexus of the Somato-Psychic Pathway. It integrates somatic afferent input with environmental demands and orchestrates physiological states that shape emotional experience, attentional capacity, and behavioral organization. Within the SPP framework, autonomic regulation is proposed as a primary developmental regulator shaping psychological organization, while acknowledging reciprocal influences across levels.

Under physiological conditions, autonomic regulation is flexible. The sympathetic and parasympathetic branches dynamically adjust to context, enabling rapid mobilization when required and an efficient return to a regulated baseline once demands subside. Myelinated vagal pathways (often described within the polyvagal framework as ventral vagal circuits), supporting social engagement and flexible autonomic regulation, contribute to emotional modulation and sustained attention, particularly during development (Porges, 2007; Thayer et al., 2012).

When somatic input is persistently dysregulating, autonomic flexibility diminishes. The system progressively enters a state conceptualized as Somato-Psychic Autonomic Dysregulation (SPAD), characterized by reduced variability, impaired recovery, and a sustained bias toward threat-oriented physiological states. SPAD represents the pathological operating mode of the Somato-Psychic Pathway.

## 5 From autonomic dysregulation to emotional and cognitive phenotype

Autonomic state profoundly shapes the tone and stability of emotional and cognitive experience. Contemporary models emphasize that emotion emerges from the brain’s interpretation of interoceptive signals rather than from isolated cortical appraisal (Craig, 2002; Seth, 2013). Within SPAD, interoceptive signalling is chronically biased toward threat. Emotional responses may become exaggerated or blunted, stress tolerance decreases, and attentional resources are increasingly diverted toward internal regulation rather than exploration or learning.

Cognitively, chronic autonomic dysregulation progressively constrains executive function, working memory, and attentional stability—sustained physiological stress tends to bias behavior toward rigidity, avoidance, or repetitive patterns (Beauchaine, 2015). Within SPP, these cognitive and behavioral phenotypes are understood as downstream expressions of autonomic state rather than as primary deficits.

## 6 Developmental trajectory: physiology, disruption, and restitution

Under physiological conditions, development proceeds from somatic structural-functional organization through autonomic stability toward emotional regulation and cognitive–social maturation. When the Somato-Psychic Pathway is disrupted early, development may slow, enter arrest, or undergo regression. Importantly, this represents functional disruption rather than irreversible damage.

Clinical observation suggests that when somatic integrity and autonomic regulation are restored, developmental functions often re-emerge in a predictable sequence. Autonomic calming precedes emotional stabilization, which in turn precedes improvements in attention, communication, and learning. This restitutive unfolding is consistent with the proposed developmental directionality and warrants systematic empirical investigation.

## 7 Somato-psychic syndrome as a clinical phenotype

Somato-Psychic Syndrome (SPS) designates the clinical phenotype arising from prolonged operation of the Somato-Psychic Pathway in its pathological mode, defined by persistent autonomic dysregulation. SPS is characterized by axial instability or rigidity, sensory hypersensitivity, autonomic lability, emotional dysregulation, attentional fluctuation, and variable developmental delay or other manifestations of developmental disruption. This phenotype spans traditional diagnostic categories and provides an explanatory framework for comorbidity and symptom variability.

SPP does not replace existing diagnoses. Instead, it provides an explanatory layer beneath them, linking heterogeneous clinical presentations to a shared somato-autonomic mechanism.

## 8 Conceptual integration and implications

The brain is not an independent generator of mind; its activity represents an adaptive response to bodily states and to environmental influences, as encountered through somatosensory inputs and their autonomic reflection. Recent refinements of Polyvagal Theory further articulate the role of brainstem-mediated autonomic state as a prerequisite for social engagement, cognitive flexibility, and recovery, providing an updated physiological context within which the Somato-Psychic Pathway can be situated without being derived from it (Porges, 2025). This view resonates with neurobiological and interoceptive frameworks that emphasize the constitutive role of bodily signals in shaping mental processes (Craig, 2002; Critchley and Harrison, 2013).

The Somato-Psychic Pathway is conceptually consonant with predictive processing frameworks (Friston, 2010; Clark, 2013), polyvagal theory (Porges, 2007, 2011), embodied cognition (Varela et al., 1991), and developmental systems theory (Thelen and Smith, 1994). This organization may be viewed as conceptually analogous to hierarchical descriptions of autonomic regulation (e.g., Porges, 2007), although the present framework is derived from developmental and clinical observations rather than phylogenetic taxonomy. Its primary contribution lies in articulating a directionally explicit, clinically grounded pathway linking somatic organization, autonomic regulation, and psychological development across both physiology and pathology.

## 9 Testable predictions and empirical roadmap

A central aim of the Somato-Psychic Pathway (SPP) framework is to generate developmentally structured, empirically tractable predictions. The model proposes a directional organization across somatic structural–functional integrity, autonomic regulation, emotional stability, and cognitive–social maturation. This section outlines candidate predictions and potential falsification criteria to clarify the scientific status of the framework.

### 9.1 Developmental ordering

SPP predicts that, in typical ontogeny, maturation of somatic organization and postural stability will precede the stabilization of autonomic flexibility, which in turn will precede the consolidation of sustained emotional regulation and higher cognitive control. Longitudinal designs assessing axial alignment, proprioceptive accuracy, and autonomic indices (e.g., heart rate variability, pupillary dynamics) alongside affective and executive measures could examine whether these domains show temporally ordered coupling consistent with the proposed developmental scaffold.

A failure to observe any systematic temporal ordering between somatic maturation, autonomic stabilization, and emotional–cognitive development would challenge the directional claim of the model.

### 9.2 Mediation by autonomic regulation

A second prediction concerns mediation. If SPP is valid, autonomic regulation should statistically mediate the relationship between somatic structural–functional integrity and downstream emotional or cognitive outcomes. For example, improvements in postural organization or reduction in persistent nociceptive input should correlate with changes in autonomic flexibility indices, which in turn should predict improvements in emotional regulation or attentional stability.

If autonomic markers do not mediate this relationship, or if somatic variables show no predictive association with autonomic indices, the central integrative role proposed for SPAD would require reconsideration.

### 9.3 Reverse-sequence clinical change

SPP further predicts that in interventional contexts, clinical improvement may frequently unfold in reverse order relative to developmental disruption. Specifically, interventions targeting somatic organization would be expected to produce measurable changes in autonomic flexibility prior to observable improvements in emotional stability and cognitive performance.

Preliminary pilot-level observations reported in abstract form (Živná and Živný, 2025) illustrate this hypothesized pattern; however, controlled interventional trials with repeated multimodal measurement are necessary to evaluate the robustness and generalizability of this sequence. If improvements in emotional or cognitive domains consistently precede measurable autonomic change in somatically targeted interventions, the proposed directional mechanism would be weakened.

### 9.4 Boundary conditions and alternative models

SPP acknowledges that bidirectional and common-cause models remain plausible alternatives. For example, primary central nervous system pathology, inflammatory processes, or genetic syndromes may independently influence both somatic organization and autonomic state. In such cases, the SPP ordering may be modified, attenuated, or overridden.

Empirical differentiation between somatic-primary, autonomic-primary, and central-primary models will require multimodal designs incorporating structural, physiological, and behavioral measures across development.

A provisional operational mapping of core constructs within the SPP framework is summarized in Table 1.

**Table 1:** Provisional operationalization of core constructs within the somato-psychic pathway framework.

Construct	Provisional definition within SPP	Candidate empirical indicators	Methodological notes
Somatic structural–functional integrity	Dynamic organization of axial and appendicular musculoskeletal alignment, mobility, and load distribution influencing afferent input	Postural symmetry analysis; segmental mobility assessment; sway metrics; movement variability indices	Age-dependent norms required; cross-sectional vs. longitudinal differentiation necessary
Distorted Proprioceptive Information (DPI)	Mismatch between peripheral somatic input and central predictive models of posture and movement	Proprioceptive matching tasks; joint position sense testing; sensorimotor integration paradigms; postural recalibration tasks	Construct-level hypothesis; requires validation studies
Persistent low-grade nociceptive drive	Chronic subthreshold nociceptive signaling influencing autonomic tone without overt pain	Quantitative sensory testing; pain threshold variability; regional mechanical sensitivity	Must differentiate from acute nociception and central sensitization syndromes
Direct peripheral autonomic modulation	Probabilistic mechanical influence on peripheral autonomic ganglia and fibers	HRV change following localized mechanical stimulation; regional sympathetic skin response	Hypothesized modulatory pathway; requires targeted physiological studies
Autonomic flexibility	Capacity of the autonomic nervous system to dynamically adjust and recover across contexts	Heart rate variability (time and frequency domain indices); respiratory sinus arrhythmia; pupillometry; recovery slope after stressor	Developmentally modulated; must control for age and baseline variability
Somato-Psychic Autonomic Dysregulation (SPAD)	Persistent autonomic bias toward reduced variability and heightened threat responsivity linked to somatic input	Reduced HRV; prolonged sympathetic dominance; impaired physiological recovery	Not a diagnostic category; functional regulatory state
Somato-Psychic Syndrome (SPS)	Clinical phenotype arising from prolonged SPAD with downstream emotional and cognitive manifestations	Composite behavioral scales; sensory reactivity profiles; attentional variability; autonomic lability markers	Provisional construct; requires differential validation against existing nosologies

## 10 Conclusion

The Somato-Psychic Pathway proposes a universal developmental trajectory through which somatic structural–functional integrity and autonomic regulation shape the emergence and stability of the mind. Operating physiologically, SPP supports emotional balance, cognitive flexibility, and developmental progression. Operating pathologically, it gives rise to Somato-Psychic Autonomic Dysregulation and the clinical phenotype of Somato-Psychic Syndrome. By integrating physiology and pathology within a single regulatory continuum, SPP offers a biologically grounded framework for understanding mental distress and neurodevelopmental disruption. Future empirical and longitudinal studies are warranted to characterize further the mechanisms, boundaries, and clinical applications of this pathway.

## Data Availability

The original contributions presented in the study are included in the article/supplementary material, further inquiries can be directed to the corresponding author.
